# Necrological

**Published:** 1897-11

**Authors:** 


### NECROLOGICAL.

Death of Dr. Nettles.—Dr. R. C. Nettles, of Marlin, was one of the best men in the medical profession. He stood high both socially and professionally, and his loss is not felt alone in Marlin and Falls county, but is shared by the entire medical profession of the State, and by all who had ever met him. His place will be hard to fill. Dr. Nettles died May 6th, 1897. We give below a sketch of his life from a Marlin paper, compiled from a history of McLennan, Falls, Bell and Coryell counties:

"Dr. Richardson Clark Nettles was born in South Carolina, April 4, 1842. His father, Rev. Abraham Nettles, was a Methodist itinerant minister, and for forty-three years a member of the South Carolina Conference. * * * His mother's name was Mary Richardson, a daughter of Mathias and Mary Richardson, of Anderson county, South Carolina. She had four children, three of whom died in early childhood; she died December 5, 1849, at the age of thirty-two.

"Upon the death of his mother, his father placed him in the care of his two maiden sisters, who lived near Summerville. Here he spent his boyhood, and received his primary education. His father valued highly the advantages of education, and gave him the best opportunities within his limited means. At the age of fourteen he was sent to the Cokesbery Conference School, and remained two years. In 1859, he entered the freshman class in Wofford College. Here he prosecuted his studies until July, 1861, having passed the required examinations for admission into the senior class, when his collegiate course was brought to a close by the outbreak of the war. The college subsequently, in 1869, unsolicited, awarded him its diploma in consideration of the time he had spent in teaching and the scientific branches pursued in his medical course of study.

"Upon the first call to arms, the youth in the schools and colleges throughout South Carolina at once girted on their armor in defense of their country, and hastened to the tented field. The same patriotic fire burned within his bosom. In August,

1861, he enlisted in Heyward's regiment, Twelve Months' Volunteers, doing service on the coast of South Carolina. In March, 1862, he went to Virginia and joined McIntosh's battery of light artillery, afterwards attached to A. P. Hill's division, Jackson's corps. With that famous command he participated in nearly every engagement fought by Lee's army, from the "Seven Days'" battle around Richmond to the second battle of Cold Harbor, in Grant's campaign against Richmond, fought June 3, 1864. The battery was then transferred to Gen. Hardie's command at Charleston, and served to the end of the war in South and North Carolina. He was color-bearer of his battery, and was severely wounded at Fredericksburg, December 13, 1862. He participated in the three days' battle of Gettysburg. On the third day his battery was in position on the center of the line of battle held by Hill's corps, opposite Cemetery Hill on the Federal line, and took part in the furious cannonade preceding the memorable charge of Pickett's division.

"He was near Greensboro, N. C., when Johnson agreed to terms of surrender. Learning of this, with a few comrades who did not wish to surrender, after the last roll-call, each taking a battery horse, they started home. He took with him, concealed about his person, the company's colors, which he returned to Miss McIntosh, the fair donor.

"Like most of his comrades who survived, he found himself utterly impoverished, but soon obtained a situation teaching school, devoting his leisure to the study of medicine. By close economy, he was enabled to matriculate at the South Carolina Medical College, and graduated in March, 1867.

"He decided to come to Texas, and located first in the northern part of Falls county, but on Christmas day, 1867, he moved to Marlin, where he has since resided and been engaged continuously in the practice of his profession. He spent three months at the Medical College and Charity Hospital in New Orleans in 1874-3; three months at Bellevue Hospital, New York, in 1878; attended the first course of lectures of the St. Louis Post-Graduate Medical College, in 1882, and spent three months at the New York Polyclinic, 1885. He was a member of the Texas State Medical Association, and of the Central Texas Medical Association. He was a member of the Falls County and the District Board of Medical Examiners.

"Dr. Nettles has always been identified with all worthy enterprises for the benefit of Marlin. He has taken special interest in the educational affairs, and was a member and president of the Board of School Trustees since the inauguration of the free school system.

"Dr. Nettles has been twice married. His first wife was Miss Lizzie Scruggs, to whom he was married May 4, 1870. She died September 5, 1875, leaving two children, Boliver Clarke and Eva Lee. His second marriage was to Miss Fannie Prendergast, daughter of Judge D. M. Prendergast, of Limestone county, on the 25th of April, 1883. Three children were born of this marriage, and they, with their mother and the older children, survive him.

"He joined the Methodist church at the age of fourteen, and has since been a devout and faithful member. He was, at the time of his death, and for a number of years prior, president of the Board of Stewards of the Marlin church.

"He was a man of positive character, and strong moral and religious convictions, and never hesitated, as a student, a soldier or a citizen, whenever it was proper, to stand for truth and morality, and religion, and law and order."

